# Decadal Analysis of Age-Adjusted Mortality Rates for Acute and Chronic Kidney Disease in Brazil, 2000-2021

**DOI:** 10.7759/cureus.61657

**Published:** 2024-06-04

**Authors:** Wilhelmina N Hauwanga, Berley Alphonse, Ifrah Akram, Albine Djeagou, Bruno Lima Pessôa, Billy McBenedict

**Affiliations:** 1 Family Medicine, Federal University of the State of Rio de Janeiro, Rio de Janeiro, BRA; 2 Neurosurgery, Fluminense Federal University, Niterói, BRA

**Keywords:** gender, age, brazil, chronic kidney disease, acute kidney injury, mortality rate

## Abstract

Introduction

Renal failure, comprising acute kidney injury (AKI) and chronic kidney disease (CKD), involves a decline or loss of kidney function. AKI is sudden and reversible, with a rapid decline in function over hours to days, while CKD involves persistent abnormalities lasting at least three months. Developing countries are seeing a rise in AKI cases, especially in critically ill patients. Globally, there's a growing occurrence and mortality rate linked to CKD.

Methods

The study used a retrospective cross-sectional design to analyze AKI and CKD mortality rates in Brazil from 2019 to 2022. Data on population and demographics, including sex and age, were obtained from the Brazilian Institute of Geography and Statistics. Mortality data for kidney diseases were sourced from the Brazilian Hospital Information System. The analysis utilized the Joinpoint Regression Program to calculate average annual percentage changes (AAPCs) and their respective 95% confidence intervals. Weighted Bayesian information criterion was used to determine the significance levels and identify the best-fitting combination of line segments and joinpoints.

Results

The study findings revealed a significant rise in AKI mortality rates for both males and females, from 2008 to 2021 (APC = 3.16; CI: 2.29 to 5.93), with higher mortality rates recorded among males compared to women over the entire study period. Analyses according to age groups showed that males between the ages 40 to 49 experienced the most rapid increase in mortality during the 2019 - 2021 period (APC = 35.41; CI: 16.72 to 46.57); meanwhile, the most rapid increase in mortality for females was observed from 2019 to 2021, and this was among those aged 30 to 39 (APC = 40.33; CI = 6.48 to 59.78). Furthermore, there was an observable upward trend in mortality related to CKD (APC = 0.70; CI: 0.41 to 1.01), with males consistently having higher mortality rates throughout the entire study period. The elderly population, both males and females, experienced the most rapid increase in CKD-related mortality, with AAPC values of 2.32 (CI: 1.82 to 2.89) for males and 1.62 (CI: 1.08 to 2.10) for females.

Conclusion

We observed a consistent increase in mortality rates from acute kidney diseases for both males and females since 2008, with males experiencing higher mortality rates overall. The study highlighted the need for further research to understand the underlying factors contributing to these trends. Additionally, interventions targeting modifiable risk factors and improving access to healthcare could help reduce mortality related to renal failure.

## Introduction

Renal failure is described as a decline or loss of kidney function. It is broadly classified as acute kidney injury (AKI) and chronic kidney disease (CKD). CKD involves the gradual loss of kidney function, while AKI is characterized by a rapid deterioration of kidney function, typically occurring within hours to days. AKI is often diagnosed alongside other acute illnesses, particularly in critically ill patients, and is associated with a focal mismatch between oxygen and nutrient delivery [1]. The primary cause of AKI is ischemia, leading to vasoconstriction, endothelial injury, and the activation of inflammatory processes [2]. The leading causes of CKD vary by setting, with hypertension and diabetes being common in developed countries, while factors like Human Immunodeficiency Virus (HIV) and exposure to toxins or heavy metals play a significant role in developing countries [3].

Historically, the etiology of AKI was divided into three categories: prerenal, renal, and postrenal. However, the combination of prerenal and renal causes of AKI is common, for example, in sepsis or cardiac surgery [4]. According to the Kidney Disease: Improving Global Outcomes (KDIGO) criteria in 2012, AKI can be diagnosed with any one of the following: creatinine increase of 0.3 mg/dL in 48 hours, creatinine increase to 1.5 times baseline within the last 7 days, or urine volume less than 0.5 mL/kg per hour for 6 hours [5]. According to the KDIGO 2012 guidelines, CKD is defined as abnormalities in kidney structure or function, present for 3 months, with health implications [6]. 

The diagnostic thresholds for CKD are an estimated glomerular filtration rate (GFR) of less than 60 mL/min/1.73 m2 and an albumin-creatinine ratio of 30 mg/g or more [6]. CKD is an important contributor to morbidity and mortality from non-communicable diseases. According to Webster et al., the primary causes of CKD vary by setting, with hypertension and diabetes being the most common causes globally, whereas factors such as HIV positive and exposure to toxins or heavy metals play a significant role in developing countries [4]. CKD has also been recognized as a risk factor for cardiovascular disease independent of other conventional risk factors for cardiovascular disease [7].

Factors such as gender, income, and geographic location play a significant role in determining the prevalence of CKD. Studies conducted on population-based samples indicate that the epidemiology of CKD varies between genders, with a higher prevalence among women, particularly in stage G3 CKD. This higher prevalence in women may be attributed to the effects of longer life expectancy on the natural decline of GFR with age, as well as the potential overdiagnosis of CKD due to the inappropriate use of GFR equations. Interestingly, there appears to be a higher proportion of men among patients initiating renal replacement therapy (RRT). This disparity could be attributed to the protective effects of estrogen in women and/or the detrimental effects of testosterone, along with unhealthy lifestyle factors, leading to a faster decline in kidney function among men compared to women [8]. A study demonstrated that renal function declined approximately twice as fast in men compared to women (−1.82 mL/min/1.73 m2 per year and −0.89 mL/min/1.73 m2 per year, respectively) [8]. This difference persisted even after adjusting for various factors and informative censoring [8]. A comprehensive study conducted in 2010 sought to assess the global prevalence and impact of CKD by analyzing data from 33 population-based studies worldwide [9]. The findings revealed that approximately 10.4% of men and 11.8% of women aged 20 and older were affected by CKD stages 1-5. This indicated a substantial burden of CKD on a global scale. The study also highlighted significant disparities in CKD prevalence based on geographic regions and income levels. In high-income countries, the prevalence was comparatively lower, with 8.6% of men and 9.6% of women affected. In contrast, in low- and middle-income countries, the prevalence was higher, affecting 10.6% of men and 12.5% of women. These findings underscored the influence of socioeconomic factors on the prevalence of CKD. Moreover, there has been a noticeable increase in the prevalence of AKI in developing countries such as Brazil. The rising burden of CKD and AKI in developing countries emphasizes the urgent need for public health interventions and strategies to prevent, detect, and manage these conditions effectively.

More than 10 million people in Brazil are estimated to be affected by some form of renal impairment. However, this condition is often overlooked, leading to delays in diagnosis and interruptions in treatment. According to census data from the Brazilian Society of Nephrology, the number of individuals receiving RRT increased by 7.7% in 2019 compared to 2018, with 45,852 people undergoing RRT [10]. The incidence of RRT was 218 patients per million, showing a 6.8% increase from the previous year [10]. Despite advancements in treatment, the crude mortality rate among Brazilian patients on hemodialysis has remained high, ranging between 15-20% per year in recent years [11]. This data underscores the need for increased awareness, early detection, and effective management of renal diseases in Brazil to improve patient outcomes and reduce the burden of renal impairment on the healthcare system.

CKD poses a significant global health challenge, especially among young adults, impacting morbidity and mortality rates. A population-based time series study using official data on mortality and hospital admissions due to CKD in individuals aged 20 to 49 years old (residents of the northern region of Brazil) in the periods 1996-2017 and 2008-2017 was conducted [12]. This study revealed that a total of 1259 deaths due to CKD occurred in individuals aged 20-49 years (young adults), living in the northern region of Brazil during the study period (1996-2017) [12]. 

Studying AKI and CKD is vital due to their significant impact on public health and individual well-being. AKI, often seen in critical illness, can lead to rapid deterioration in kidney function and is associated with high mortality rates, while CKD is a progressive condition characterized by the gradual loss of kidney function, leading to complications such as cardiovascular disease and end-stage renal disease. However, in Brazil, research focusing on CKD and AKI is limited. To bridge this gap, we conducted a population-based time series study, analyzing official data on age-adjusted mortality rates (AAMR) from 2000 to 2021. Furthermore, we aimed to evaluate the impact of factors such as age and gender on the age-adjusted mortality rate, with the goal of providing policymakers with valuable insights for developing targeted interventions.

## Materials and methods

Study design and data collection

We carried out a descriptive, time series study using kidney disease mortality data (AKI and CKD) from Brazil, from 2000 to 2021. Mortality data was obtained from the Brazilian Hospital Information System (DATASUS), under the Sistema Único de Saúde (SUS), a Brazilian Unified Health System. DATASUS gathers information from all hospitalizations reimbursed by the SUS, which includes approximately 80% of the Brazilian population. The tenth edition of the International Classification of Diseases (ICD 10) was used for records selection, and those coded N17 to N19 were included (which correspond to Acute renal failure, i.e., N17; CKD, i.e., N18, and unspecified kidney failure, i.e., N19).

Population estimates and mortality rates

Population and demographic data, including details regarding sex and age, were sourced from the population estimates provided by the Brazilian Institute of Geography and Statistics within its Demographic and Socioeconomic Information Section. We computed the age-standardized mortality rate for acute and CKD across all age groups. The mortality rates were expressed per 100,000 individuals, and the age-adjusted rates were determined through direct standardization, employing the world standard population as a reference [13].

Time series analysis

To assess temporal trends in kidney diseases mortality rates, the average annual percentage changes (AAPCs) along with their respective 95% confidence intervals were computed employing joinpoint regression [14]. The AAPC was derived as a geometrically weighted mean of different annual percentage change values acquired from the regression analysis. Joinpoint Regression Program (Version 5.0.2., May 2023) is a trend analysis software developed by the Statistical Research and Applications Branch of the National Cancer Institute (Bethesda, MD, United States) for the analysis of data from the Surveillance Epidemiology and End Results Program. Weighted Bayesian information criterion was used to test for the level of significance and determine the best-fitting combination of line segments and joinpoints [14]. This examination aimed to investigate variations in both acute and chronic disease mortality trends across categories such as age and sex.

## Results

Acute kidney failure by gender

**Figure 1:**
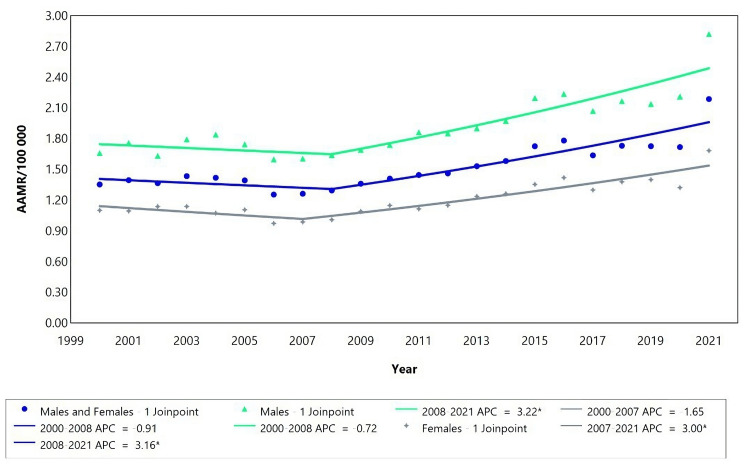
Mortality rate trend by acute kidney failure using Joinpoint analysis for the variable sex for the period 2000 to 2021. AAMR = Age-adjusted mortality rate; APC = Annual percent change.

The mortality rate due to acute kidney failure (AKF) displayed a downward trend for both "males and females" from 2000 to 2008, followed by an upward trend from 2008 to 2021, with a significant annual percentage change (APC) of 3.16. The data analysis for AKF between 2000 and 2008 consistently showed a decreasing trend, as indicated by the APC values (Table 1). In contrast, the data for AKF between 2008 and 2021 demonstrated an increasing trend, supported by a significant APC (Table 1). Furthermore, the decrease in mortality rates related to AKF was more notable among females (AAPC=1.43) compared to males (AAPC=1.70). These trends are visually depicted in Figure 1.

**Table 1:** Mortality rate trend by acute kidney failure using Joinpoint analysis for the variable sex for the period 2000 to 2021. *Significant at P < 0.05 level. AAMR = Age-adjusted mortality rate; AAPC = Average annual percent change; APC = Annual percent change.

Gender	Period (years)	APC (95% CI)	AAPC (95% CI)
Males and Females	2000 to 2008	-0.91 (-6.96 to 1.11)	1.59* (1.00 to 2.26)
Males and Females	2008 to 2021	3.16* (2.29 to 5.93)	
Males	2000 to 2008	-0.72 (-8.24 to 1.45)	1.70* (1.03 to 2.47)
Males	2008 to 2021	3.21* (2.28 to 7.29)	
Females	2000 to 2007	-1.65 (-7.40 to 0.61)	1.43* (0.89 to 2.03)
Female	2007 to 2021	3.00* (2.26 to 4.53)	

The age-adjusted mortality rate for “male and female combined” ranged from 1.30 deaths/100,000 to 1.70 deaths/100,000 inhabitants (Figure 1). The age-adjusted mortality rate was more pronounced in males, ranging from an AAMR of 1.80/100,000 inhabitants in 2000 to 2.40/100,000 in 2021. Females on the other hand had recorded the lowest age-adjusted mortality rate ranging from 1.10/100,000 in 2000 to 1.40/100,000 in 2021 as shown in Figure 1. Overall, the AKF mortality rate showed an increasing trend, with the highest values recorded in 2021. In addition, the mortality rates were higher in males than in females.

Acute kidney failure by age

Males (Children and Adults)

Between 2019 and 2021, three age groups demonstrated a significant increase in APC, as detailed in Table 2. The highest APC was observed among males aged 40-49, followed by 50-59, and then 30-39. In contrast, the 20-29 age group experienced a decrease in APC of -1.49 between 2000 and 2019. Additionally, there was an increase in APC among males aged 60-69 and 80 and above (APC: 3.64; 95% CI: 2.56 to 7.35, APC: 3.13; 95% CI: 2.07 to 8.30, respectively) in the years 2007-2021. Interestingly, the 70-79 age group did not exhibit any joinpoints where the APC value was equal to the AAPC of 2.26 (95% CI: 1.68 to 2.90).

**Table 2:** Acute kidney failure mortality rate trends for males, using Joinpoint analysis for the period 2000 to 2021. *Significant at P < 0.05 level. AAMR = Age-adjusted mortality rate; AAPC = Average annual percent change; APC = Annual percent change.

Age groups	Period (years)	APC (95% CI)	AAPC (95% CI)
0 - 4 years	2000 to 2021	1.23 (-0.75 to 3.40)	1.23 (-0.75 to 3.40)
5 - 9 years	2000 to 2015	-1.34 (-7.40 to 1.25)	-2.38* (-5.37 to -0.49)
5 - 9 years	2015 to 2018	32.57* (7.75 to 50.54)	
5 - 9 years	2018 to 2021	-31.85* (-57.43 to -18.65)	
10 - 14 years	2000 to 2021	1.23 (-1.35 to 4.20)	1.23 (-1.35 to 4.20)
15 - 19 years	2000 to 2021	0.81 (-0.93 to 2.66)	0.81 (-0.93 to 2.66)
20 - 29 years	2000 to 2019	-1.49* (-6.29 to -0.043)	0.20 (-1.46 to 1.13)
20 - 29 years	2019 to 2021	17.75 (-0.89 to 30.61)	
30 - 39 years	2000 to 2010	-3.00 (-11.01 to 2.61)	1.98* (0.78 to 2.85)
30 - 39 years	2010 to 2019	3.34 (-5.03 to 7.39)	
30 - 39 years	2019 to 2021	23.51* (6.24 to 34.76)	
40 - 49 years	2000 to 2019	0.20 (-0.71 to 0.95)	3.11* (2.23 to 3.86)
40 - 49 years	2019 to 2022	35.41* (16.72 to 46.57)	
50 - 59 years	2000 to 2019	0.87 (-0.80 to 1.79)	3.17 (1.72 to 3.99)
50 - 59 years	2019 to 2021	27.96* (6.14 to 38.36)	
60 - 69 years	2000 to 2007	-2.60 (-12.19 to 0.90)	1.52* (0.73 to 2.52)
60 - 69 years	2007 to 2021	3.64* (2.56 to 7.35)	
70 - 79 years	2000 to 2021	2.26* (1.68 to 2.90)	2.26* (1.68 to 2.90)
80 years and above	2000 to 2007	-0.63 (-9.89to 2.21)	1.86* (1.15 to 2.78)
80 years and above	2007 to 2021	3.13* (2.07 to 8.30)	

**Figure 2:**
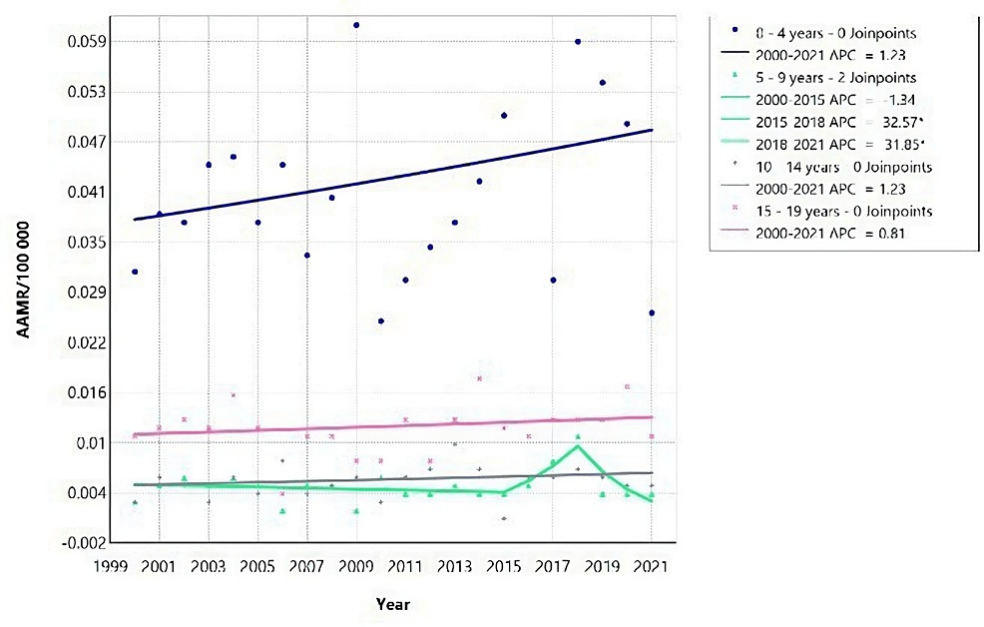
Acute kidney failure mortality rate trends for male children, using Joinpoint analysis for the period 2000 to 2021. AAMR = Age-adjusted mortality rate; APC = Annual percent change.

Between 2015 and 2018, there was a significant rise in mortality rates among males aged 5-9 (Figure 2), with an APC of 32.57 (95% CI: 7.75 to 50.54), followed by a decrease in 2018-2021, with an APC of -31.85 (95% CI: -57.43 to -18.65) (Table 2). Despite these fluctuations, there was an overall decrease in average mortality for this age group over the entire study period, indicated by a negative AAPC. 

**Figure 3:**
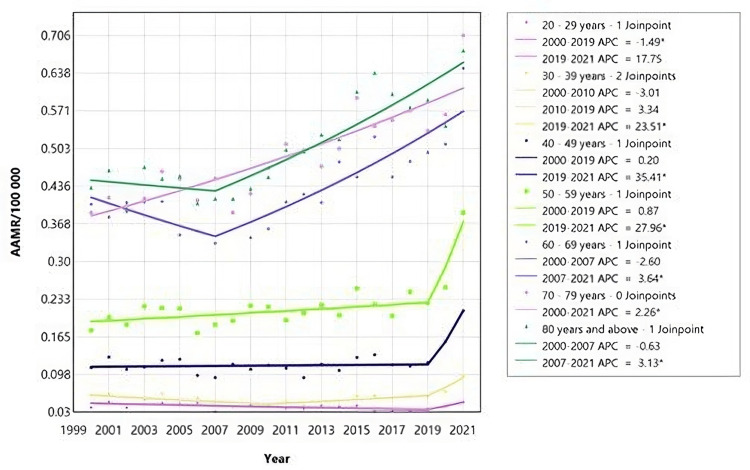
Acute kidney failure mortality rate trends for males male adults, using Joinpoint analysis for the period 2000 to 2021. AAMR = Age-adjusted mortality rate; APC = Annual percent change.

Throughout the study period (2000-2021), males in the age group “5-9” demonstrated a decrease in AAMR. On the other hand, males of age groups “30-39”, “40-49”, “50-59”, “60-69”, “70-79”, “80 and above” had an increase in mortality rate, as seen on figure 3: with the highest increase in the “50-59” age group and least increase in “60-69” age group (Figure 3, Table 2). 

 Females (Children and Adults)

**Figure 4:**
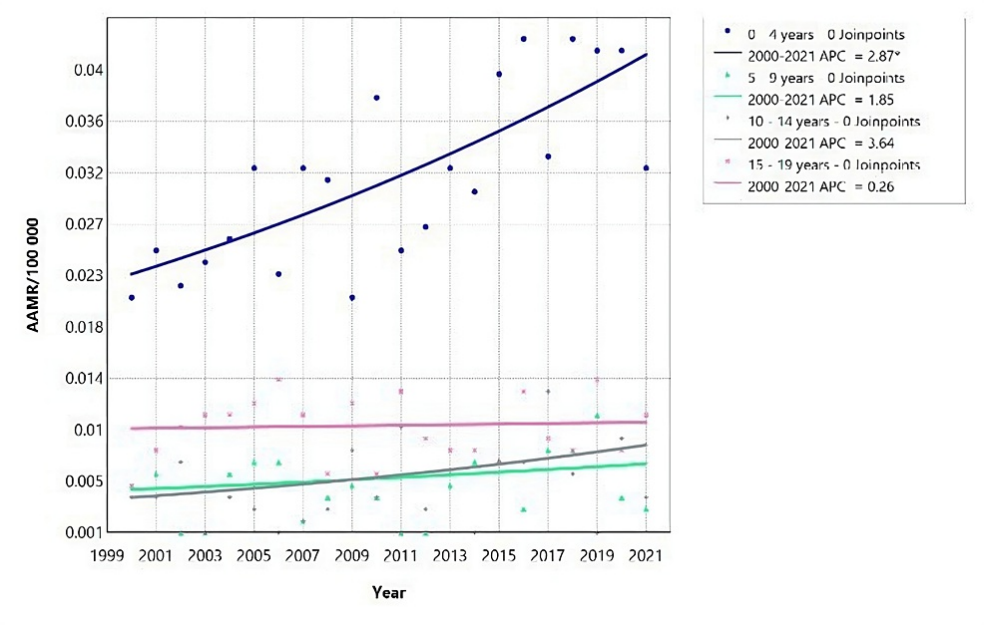
Acute kidney failure mortality rate trends for young females, using Joinpoint analysis for the period 2000 to 2021. Between 2000 and 2021, the AAMR for the age group 0-4 years increased with an APC of 2.87 (95% CI: 1.47 to 4.53). However, for the age group 5-19 years, there was no significant change in the APC during the same period. AAMR = Age-adjusted mortality rate; APC = Annual percent change.

The mortality due to AKF among females in Brazil from 2000 to 2021 showed an increase with age (Figure 4, Table 3). Notably, for children aged 0-4 years, there was a significant increase in the APC of 2.87* (95% CI: 1.47-4.53).

**Table 3:** Acute kidney failure mortality rate trends for females, using Joinpoint analysis for the period of 2000 to 2021. *Significant at P < 0.05 level. AAMR = Age-adjusted mortality rate; AAPC = Average annual percent change; APC = Annual percent change.

Age groups	Period (years)	APC (95% CI)	AAPC (95% CI)
0 - 4 years	2000 to 2021	2.87* (1.47 to 4.53)	2.87* (1.47 to 4.53)
5 - 9 years	2000 to 2021	1.85 (-2.47 to 7.29)	1.85 (-2.47 to 7.29)
10 - 14 years	2000 to 2021	3.63 (-0.27 to 9.15)	3.63 (-0.27 to 9.15)
15 - 19 years	2000 to 2021	0.25 (-2.03 to 2.64)	0.25 (-2.03 to 2.64)
20 - 29 years	2000 to 2021	-1.88* (-3.52 to -0.36)	-1.88* (-3.52 to -0.36)
30 - 39 years	2000 to 2019	-0.79 (-3.31 to 0.53)	2.53* (0.47 to 3.76)
30 - 39 years	2019 to 2021	40.33* (6.48 to 59.78)	
40 - 49 years	2000 to 2021	1.05 (-0.18 to 2.33)	1.05 (-0.18 to 2.33)
50 - 59 years	2000 to 2008	-2.91 (-13.11 to 6.97)	2.04* (0.81 to 2.98)
50 - 59 years	2008 to 2019	2.81 (-6.93 to 6.42)	
50 - 59 years	2019 to 2021	19.54* (3.98 to 29.92)	
60 - 69 years	2000 to 2007	-2.76 (-15.17 to 1.50)	1.71* (0.78 to 2.98)
60 - 69 years	2007 to 2021	4.03* (2.62 to 10.54)	
70 - 79 years	2000 to 2021	2.29* (1.66 to 3.02)	2.29* (1.66 to 3.02)
80 years and above	2000 to 2007	-2.55 (-9.60 to 0.02)	0.59 (-0.23 to 1.24)
80 years and above	2007 to 2016	4.31* (2.77 to 12.48 )	
80 years and above	2016 to 2021	-1.47 (-8.27 to 1.28)	

**Figure 5:**
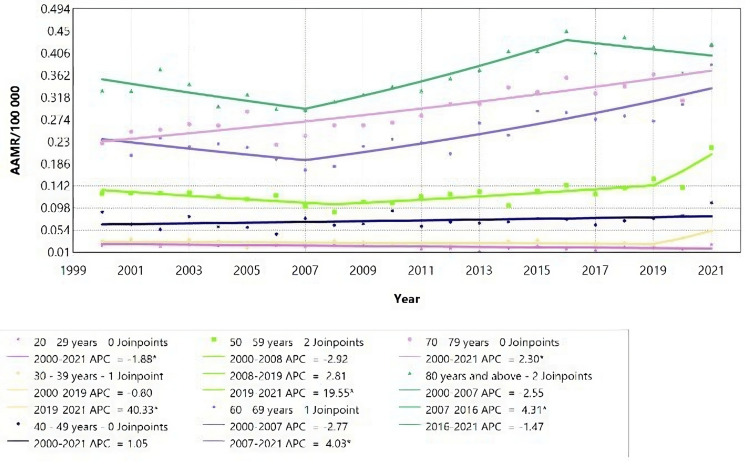
Acute kidney failure mortality rate trends for female adults, using Joinpoint analysis for the period 2000 to 2021. AAMR = Age-adjusted mortality rate; APC = Annual percent change.

Interestingly, there were no joinpoints identified for females in the age groups 0-29 years, 40-49 years, and 70-79 years, indicating a consistent trend in mortality rates across these age groups (Figure 5, Table 3)

According to Figure 5, female adults aged 20-29 years showed a decrease in AAMR with an APC of -1.88 (95% CI: -3.52 to -0.36) for the period 2000 to 2021 (Table 3). In contrast, females aged 70-79 years experienced an increase in AAMR with an APC of 2.29 (95% CI: 1.66 to 3.02) over the same period. Additionally, there was an increase in AAMR for females aged 30-39 years and 50-59 years, indicated by an APC of 40.33 (95% CI: 6.48 to 59.78) and APC of 19.55 (95% CI: 3.98 to 29.92), respectively. From 2007 to 2021, females aged 60-69 years showed a significant increase in AAMR with an APC of 4.03 (95% CI: 2.62 to 10.54). Similarly, females aged 80 years and above had an increase in AAMR between 2007 and 2016, with an APC of 4.31 (95% CI: 2.77 to 12.48) (Figure 5).

Chronic kidney failure by gender

**Figure 6:**
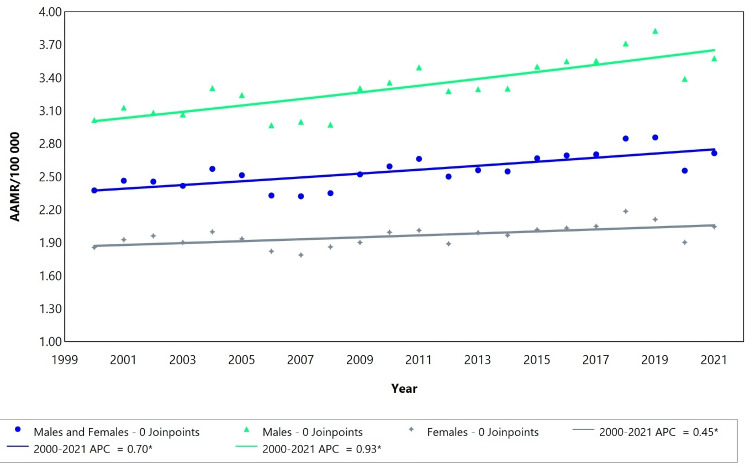
Chronic kidney failure for both males and females regardless of age. AAMR = Age-adjusted mortality rate; APC = Annual percent change.

The mortality rate related to CKD showed a steady increase for both males and females independently, as well as when considered collectively, from 2000 to 2021 (Figure 6).

The combined cohort of males and females displayed a trend with no joinpoints, indicating a consistent rise in mortality, with an estimated APC of 0.70 (95% CI: 0.41 to 1.01) (Table 4). Similarly, significant positive trends were noted for males (APC = 0.93; 95% CI: 0.58 to 1.31) and females (APC = 0.45; 95% CI: 0.19 to 0.74) when analyzed separately, also with no joinpoints, suggesting a continuous increase in mortality over the entire study period (Table 4).

**Table 4:** APC and AAPC for chronic kidney failure based on gender. *Significant at P < 0.05 level. AAPC = Average annual percent change; APC = Annual percent change.

Gender	Period (year)	APC (95% CI)	AAPC (95% CI)
Males and Females	2000 to 2021	0.70* (0.41 to 1.01)	0.70* (0.41 to 1.01)
Males	2000 to 2021	0.93* (0.58 to 1.31)	0.93* (0.58 to 1.31)
Females	2000 to 2021	0.45* (0.19 to 0.74)	0.45* (0.19 to 0.74)

Figure 6 illustrates the increasing trend of AAMR by gender, with the highest rates observed in males. In 2000, the AAMR for males was 3.00/100,000, rising to 3.60/100,000 by 2021. For the combined cohort of males and females, the AAMR was 2.40/100,000 in 2000, increasing moderately to 2.80/100,000 in 2021. The AAMR for females also showed an increase, from 1.90 deaths/100,000 in 2000 to 2.20 deaths/100,000 in 2021.

Chronic kidney failure by age and gender

Males

The mortality rate for males with CKD showed a steady evolution from 2000 to 2021, with no joinpoint observed for most age groups. However, one joinpoint was observed for the 20-29 years, 30-39 years, and 70-79 years age groups (Table 5). The change in mortality rate varied across age groups, with some displaying an increasing trend while others showed a decreasing trend (Figure 5).

**Table 5:** Trends in chronic kidney disease-related mortality among Brazilian men from 2000 to 2021. *Significant at P < 0.05 level. AAMR = Age-adjusted mortality rate; AAPC = Average annual percent change; APC = Annual percent change.

Age groups	Period (years)	APC (95% CI)	AAPC (95% CI)
0 - 4 years	2000 to 2021	2.82* (0.02 to 6.30)	2.82* (0.02 to 6.30)
5 - 9 years	2000 to 2021	-4.02* (-7.86 to -0.78)	-4.02* (-7.86 to -0.78)
10 - 14 years	2000 to 2021	-1.96 (-6.06 to 1.82)	-1.96 (-6.06 to 1.82)
15 - 19 years	2000 to 2021	0.19 (-1.42 to 1.81)	0.19 (-1.42 to 1.81)
20 - 29 years	2000 to 2013	-2.58* (-12.68 to -0.52)	-0.49 (-1.88 to 0.75)
20 - 29 years	2013 to 2021	2.99 (-0.81 to 19.91)	
30 - 39 years	2000 to 2019	-0.36 (-2.89 to 0.32)	0.76 (-0.27 to 1.35)
30 - 39 years	2019 to 2021	11.99* (0.23 to 19.21)	
40 - 49 years	2000 to 2021	-0.54 (-1.31 to 0.23)	-0.54 (-1.31 to 0.23)
50 - 59 years	2000 to 2021	-0.46 (-0.92 to 0.001)	-0.46 (-0.92 to 0.001)
60 - 69 years	2000 to 2021	0.85* (0.38 to 1.37)	0.85* (0.38 to 1.37)
70 - 79 years	2000 to 2019	1.53* (0.07 to 11.81)	0.84* (0.04 to 2.21)
70 - 79 years	2019 to 2021	-5.47 (-13.34 to 1.88)	
80 years and above	2000 to 2021	2.32* (1.82 to 2.89)	2.32* (1.82 to 2.89)

**Figure 7:**
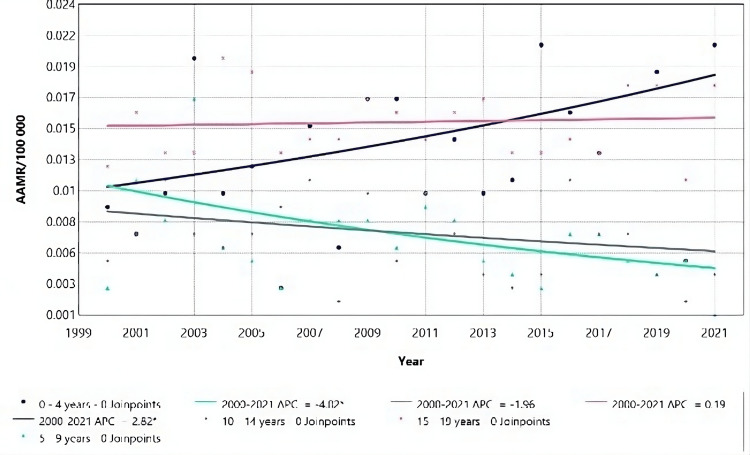
Chronic kidney failure mortality rate trends for male children, using Joinpoint analysis for the period 2000 to 2021. AAMR = Age-adjusted mortality rate; APC = Annual percent change.

**Figure 8:**
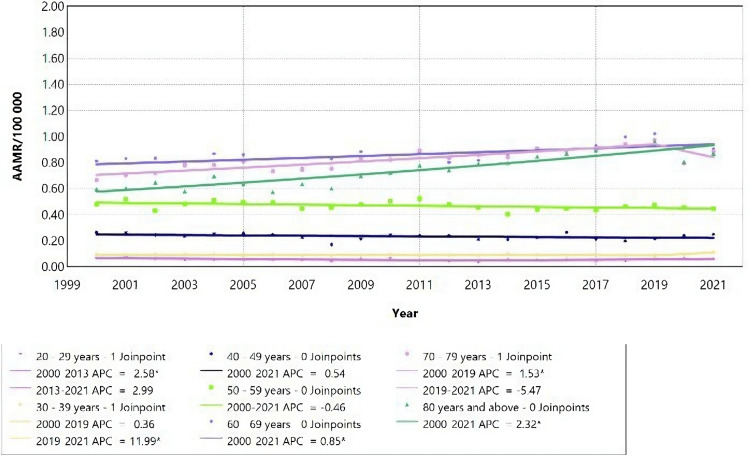
Chronic kidney failure mortality rate trends for male adults, using Joinpoint analysis for the period 2000 to 2021. AAMR = Age-adjusted mortality rate; APC = Annual percent change.

For individuals aged 20 to 29 years, the mortality rate steadily decreased from 2000 to 2013 (APC = 2.58; 95% CI: -12.68 to -0.52), followed by a non-significant increase until 2021. Among males aged 30 to 39 years, there was a rapid increase in mortality rate from 2019 to 2021 (APC = 11.99; 95% CI: 0.23 to 19.21); however, the decrease observed from 2000 to 2019 was not statistically significant. For those aged 70 to 79 years, the mortality rate increased significantly from 2000 to 2019 (APC = 1.53; 95% CI: 0.07 to 11.81), with no significant change from 2019 to 2021. The mortality rate related to CKD increased significantly from 2000 to 2021 for those aged 0 to 4 years, 60 to 69 years, 70 to 79 years, and those aged 80 and above (Figures 7, 8). However, there was a significant decrease in mortality rate for those aged 5 to 9 years (Figure 8).

Females

**Figure 9:**
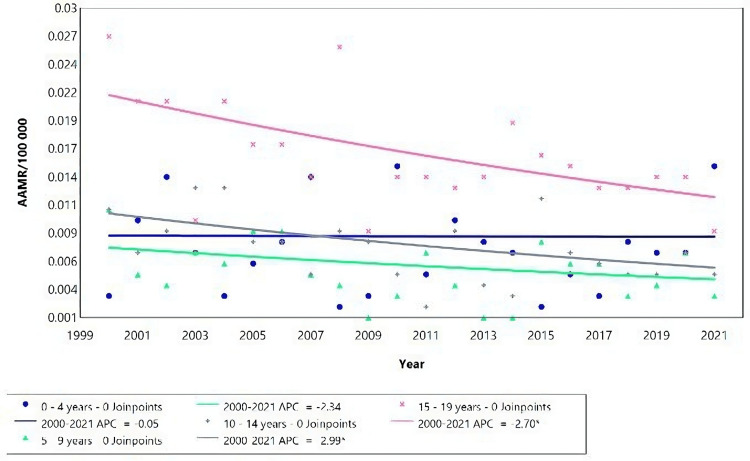
Chronic kidney failure mortality rate trends for young females, using Joinpoint analysis for the period 2000 to 2021. AAMR = Age-adjusted mortality rate; APC = Annual percent change.

**Figure 10:**
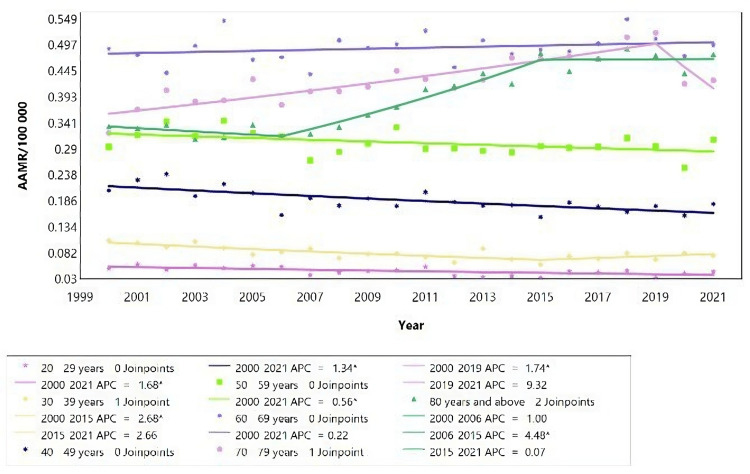
Chronic kidney failure mortality rate trends for female adults, using Joinpoint analysis for the period 2000 to 2021. AAMR = Age-adjusted mortality rate; APC = Annual percent change.

The overall rate of mortality related to CKD decreased significantly from 2000 to 2021 for females aged 10 to 59 years (Figures 9, 10). Conversely, it increased for those aged 80 and above. There was no significant change in the mortality rate for females younger than 10 and those aged 60 to 79 years, as indicated by the AAPC values presented in Table 6.

**Table 6:** Trend in chronic kidney disease-related mortality amongst Brazilian females from 2000 to 2021. *Significant at P < 0.05 level. AAPC = Average annual percent change; APC = Annual percent change.

Age groups	Period (years)	APC (95% CI)	AAPC (95% CI)
0 - 4 years	2000 to 2021	-0.05 (-4.64 to 4.77)	-0.05 (-4.64 to 4.77)
5 - 9 years	2000 to 2021	-2.34 (-6.78 to 1.54)	-2.34 (-6.78 to 1.54)
10 - 14 years	2000 to 2021	-2.99* (-5.93 to -0.49)	-2.99* (-5.93 to -0.49)
15 - 19 years	2000 to 2021	-2.70* (-4.49 to 1.08)	-2.70* (-4.49 to 1.08)
20 - 29 years	2000 to 2021	-1.68* (-2.98 to -0.46)	-1.68* (-2.98 to -0.46)
30 - 39 years	2000 to 2015	-2.68* (-8.61 to -1.57)	-1.19* (-2.34 to -0.19)
30 - 39 years	2015 to 2021	2.66 (-1.44 to 16.57)	
40 - 49 years	2000 to 2021	-1.34* (-2.00 to -0.70)	-1.34* (-2.00 to -0.70)
50 - 59 years	2000 to 2021	-0.56* (-1.07 to -0.04)	-0.56* (-1.07 to -0.04)
60 - 69 years	2000 to 2021	0.22 (-0.19 to 0.64)	0.22 (-0.19 to 0.64)
70 - 79 years	2000 to 2019	1.74* (1.34 to 2.65)	0.63 (-0.07 to 1.39)
70 - 79 years	2019 to 2021	-9.32 (-15.99 to 0.01)	
80 years and above	2000 to 2006	-1.00 (-7.06 to 1.29)	1.62* (1.08 to 2.10)
80 years and above	2006 to 2015	4.48* (3.41 to 9.99)	
80 years and above	2015 to 2021	0.07 (-3.20 to 1.68)	

For females aged 30 to 39 years, the mortality rate decreased from 2000 to 2015 (APC = -2.68; 95% CI: -8.61 to -1.57), followed by a subsequent increase that was not statistically significant. Females aged 70 to 79 years experienced an initial increase in mortality rate from 2000 to 2019 (APC = 1.74; 95% CI: 1.34 to 2.65), followed by a non-significant change in trend (Table 6). Among females aged 80 years and above, the mortality rate increased from 2006 to 2015 (APC = 4.48; 95% CI: 3.41 to 9.99), with no statistically significant changes in trends observed before or after this period (Figure 10).

There was a consistent change in CKD-related mortality rate for most age groups, indicated by the absence of joinpoints, except for the age groups "30-39", "70-79", and "80 years and above", which had at least one Joinpoint (Table 6, Figure 9, and Figure 10). The most significant decrease in mortality rate was observed in young girls aged 15 to 19, with the rate dropping from an average of 0.027 deaths per 100,000 in 2000 to 0.009 deaths per 100,000 in 2021. Among adults, the most substantial drop in mortality rate was seen in those aged 30 to 39 years, decreasing from an average of 0.107 deaths per 100,000 in 2000 to an average of 0.077 deaths per 100,000 in 2021. Conversely, the highest increase was observed among those aged 80 and above, with an AAMR rising from 0.335 per 100,000 in 2000 to 0.479 in 2021.

## Discussion

This study investigated the mortality trends of Acute and CKDs in Brazil from 2000 to 2021, focusing on gender and age as variables. According to the 2010 Global Burden of Disease study, CKD rose from being the 27th to the 18th leading cause of total deaths worldwide between 1990 and 2010 [15]. CKD, a progressive condition affecting over 10% of the global population, equating to more than 800 million people, is more prevalent in older individuals, females, racial minorities, and those with co-existing conditions like diabetes and hypertension [15]. This disease places a significant burden on low and middle-income countries, which often lack adequate healthcare facilities. The high number of affected individuals and the serious consequences of these diseases underscores the urgent need for improved prevention and treatment strategies. Additionally, the presence of pre-existing CKD significantly impacts the outcomes of AKI. A retrospective study demonstrated that individuals with CKD are not only at higher risk of AKI but also experience longer durations of RRT and increased in-hospital resuscitation rates [16]. The in-hospital mortality rate for AKI patients, particularly those requiring dialysis, ranges from 30-50%. Negative prognostic factors for AKI outcomes include age, oliguria, multi-organ dysfunction, and hypotension [17].

Acute kidney failure 

In our study, the mortality rate due to AKF generally decreased for both sexes between 2000 and 2008, but increased during 2008-2021, interestingly, the mortality rate due to AKF decreased more in females than in males over the entire study period. The same observation was made by Neugarten's team; in their study, men were 2.19 times more affected [18]. A study on gender differences in kidney function suggests that the influence of sex hormones could be the basis for the sex ratio observed in kidney-related issues [19]. This phenomenon may also be explained by the "male-female health-survival paradox", where biopsychosocial factors are studied to understand why men tend to die more than women. Men often engage in higher-risk behaviors such as alcohol consumption and smoking, which can increase their risk of developing hypertension and subsequent kidney failure. Additionally, women may perceive disease differently from men, actively seeking follow-up and preventive care. Women also tend to have more support networks, which can positively impact their health outcomes [20]. However, the mortality rate of AKF in women aged over 65 years was found to be higher than that in men of the same age group. This difference is attributed to structural changes and comorbidities caused by aging, as well as a decrease in estrogen levels [21]. Despite AKF being more common in males than females, a study by Loutradis et al. showed no difference in the mortality rate between the two sexes [22].

Our study indicates that mortality in patients with AKI increased in newborns and infants aged between 0-4 years, with mortality rates increasing with age. There is a correlation between prematurity and mortality in newborns with acute renal failure [23]. Age and sex are significant risk factors that can influence mortality in patients with acute renal failure. However, other factors must also be considered, including the recurrence of acute renal failure, the classification of acute renal failure, admission to the hospital's intensive care unit, the time between diagnosis and treatment, and the need to initiate dialysis [24]. These additional factors play a crucial role in determining the outcomes and management of acute renal failure in patients, highlighting the complexity of this condition and the importance of a comprehensive approach to its treatment.

Chronic kidney failure

Regarding CKD, our study found a gradual and persistent increase in CKD-related mortality rates for both males and females since 2000, with males being more affected than females. Elderly individuals showed a higher mortality rate compared to other age groups for both genders. This is in agreement with de Souza Anacleto et al. study which investigated the factors associated with mortality from renal failure between 2009 and 2019 [25]. The study identified several key factors, including the presence of chronic-degenerative diseases, age above 50 years or below a year, a low level of education, and being native to the South and Southeast regions of Brazil. Other risk factors include diabetes, hypertension, and limited access to RRT [26]. These findings highlight the complex interplay of socioeconomic, demographic, and geographic factors that contribute to mortality from renal failure in Brazil. Understanding these factors is crucial for developing targeted interventions to reduce mortality rates and improve outcomes for individuals with renal failure. Similar to our findings, Gouvêa et al. reported a rising trend in CKD mortality in Brazil from 2009 to 2020, with sociodemographic discrepancies [27]. 

Despite national strategies, the quality of care for CKD patients in Brazil remains inadequate, with limited access to essential medications, and health products, and a shortage of specialists [28]. Furthermore, many countries, including Brazil, lack explicit measures in their national policies and strategies to improve the population's knowledge of CKD prevention, screening, and treatment [29]. This underscores the importance of governments implementing measures to address these obstacles and improve the care and outcomes for CKD patients. This highlights the ongoing challenges in addressing CKD-related mortality in Brazil and the need for continued efforts to improve prevention and treatment strategies.

Our study revealed a gradual increase in the mortality rate related to CKD for both Brazilian males and females. However, the rate was consistently higher for males, and they also experienced a more rapid increase in mortality over time (Figure 4). This gender disparity in CKD-related mortality is consistent with findings from the study by Paciej-Gołębiowska et al. [30]. In contrast, other studies have reported a higher mortality rate among women with CKD compared to men, with one of the major reasons being unequal access to healthcare, which tends to favor men [31]. These findings highlight the need for further research to understand the underlying factors contributing to gender disparities in CKD-related mortality and to develop strategies to address them.

Several public policies aimed at preventing and managing kidney diseases have been implemented in Brazil in recent years [32]. In 2004, the National Policy for Attention to Patients with CKD was established through Ordinance No. 1.168/2004 [33]. This was followed by the release of guidelines for the Clinical Prevention of Cardiovascular, Cerebrovascular, and CKD by the Ministry of Health in 2006. These guidelines recommended early screening in primary care for at-risk groups, such as individuals with arterial hypertension, diabetes mellitus, and those with a family history of CKD. In 2014, the Ministry of Health issued Ordinance No. 389/2014, which outlined the Clinical Guidelines for the Care of Patients with CKD in the Sistema Único de Saúde. Furthermore, the Federal Government developed the Strategic Action Plan to Combat Chronic Noncommunicable Diseases in Brazil 2011-2022, which has recently been updated for the period 2021-2030. These policies highlight the importance of controlling and treating the risk factors associated with CKD as a primary strategy for preventing the disease [33].

Limitations

Although secondary data can be advantageous and cost-effective, it does come with inherent limitations that need to be acknowledged. Firstly, there may be unreported cases leading to incomplete data, as well as concerns regarding the reliability and accuracy of the data, particularly in terms of assigning appropriate ICD-10 codes to mortality cases. Another limitation of this study was the challenge of directly comparing outcomes with those of other researchers due to the inclusion of multiple ICD-10 codes. Additionally, the study only analyzed age and sex as variables, and future studies could consider investigating additional variables such as state and region of residence. Lastly, the study's exclusive focus on mortality patterns of kidney diseases without exploring the causal factors behind these trends is another limitation that should be noted.

Strengths

Despite the mentioned drawbacks, our study has several strengths that contribute to its significance. Firstly, it is the first of its kind in Brazil, providing unique insights into the trends of acute and CKD mortality in the country. The study's extensive time period, spanning from 2000 to 2021, and its wide age range of patients (0 to 80+ years) are notable strengths. Additionally, the use of data from the national database ensures that the findings are representative of the Brazilian population. The substantial sample size further enhances the statistical power of the study, allowing for more robust conclusions to be drawn. The study's ability to identify a significant increase in mortality due to acute and CKD in recent years highlights the urgency of addressing these issues. Furthermore, the longitudinal analysis of mortality trends enables the identification of variations across different subgroups, providing valuable insights for targeted interventions. Overall, the study's findings are crucial for informing healthcare policies and practices aimed at reducing the burden of kidney diseases in Brazil and improving outcomes for affected individuals.

## Conclusions

The findings of our study highlight concerning trends in acute and chronic kidney disease (CKD) mortality rates in Brazil. We observed a consistent increase in mortality rates from acute kidney diseases for both males and females since 2008, with males experiencing higher mortality rates overall. Interestingly, there was a decrease in mortality among males aged 5-9 years, but an increase among males aged 30-80 years and older. Similarly, female children aged 0-4 years and adults aged 30 years and above also showed a significant increase in mortality from acute kidney disease. CKD-related mortality rates have also risen substantially from 2000 to 2021, with males being more affected than females. Elderly individuals, in particular, demonstrated higher mortality rates from CKD. These findings underscore the need for targeted public health initiatives to address the age and gender disparities in kidney disease susceptibility and mortality, with a focus on enhancing prevention and treatment strategies.
